# The perception of aquaculture on the Swedish West Coast

**DOI:** 10.1007/s13280-017-0945-3

**Published:** 2017-09-22

**Authors:** Jean-Baptiste E. Thomas, Jonas Nordström, Emma Risén, Maria E. Malmström, Fredrik Gröndahl

**Affiliations:** 1 Industrial Ecology, Department of Sustainable Development, Environmental Science and Engineering (SEED), KTH Royal Institute of Technology, Teknikringen 34, 10044 Stockholm, Sweden; 2 Department of Food and Resource Economics, University of Copenhagen, Rolighedsvej 25, 1958 Frederiksberg C, Denmark; 3 Agrifood Economics Centre, Lund University, Lund, Sweden; 4 Present Address: Sweco Environment AB, Gjörwellsgatan 22, 112 60 Stockholm, Sweden

**Keywords:** Aquaculture, Bioeconomy, Blue growth, Macroalgae, Perception survey, Social acceptability

## Abstract

Efforts are on the way on the Swedish West Coast to develop the capacity for cultivation of marine resources, notably of kelps. Given that this is a region of great natural and national heritage, public opposition to marine developments has been identified as a possible risk factor. This survey thus sought to shed light on awareness levels, perceptions of different types of aquaculture and on reactions to a scenario depicting future aquaculture developments on the West Coast. When asked about their general opinions of aquaculture, respondents tended to be favourable though a majority chose neutral responses. On the whole, respondents were favourable to the depicted scenario. Finally, it was found that the high-awareness group tended to be more supportive than the low or medium-awareness groups, hinting at the benefits of increasing awareness to reduce public aversion and to support a sustainable development of aquaculture on the Swedish West Coast.

**Electronic supplementary material:**

The online version of this article (doi:10.1007/s13280-017-0945-3) contains supplementary material, which is available to authorized users.

## Introduction

There is a rising tide of interest in the cultivation of seaweed biomass in Europe. Cultivated seaweed provide distinguished advantages over other cultivated biomasses: they require little or no arable land, fertilisers or fresh water (Subhadra and Edwards 2010; John et al. 2011; Wei et al. 2013) while providing a variety of other ecosystem services, including nutrient bioremediation (Chung et al. 2002) and possibly habitat provision (Phillips 1990). Seaweed biomass shows promising potential as a material in the production of biofuels, fertiliser, materials, chemicals, feed and food (Jung et al. 2013; van Hal et al. 2014; Chapman et al. 2015; Pechsiri et al. 2016; Tayyab et al. 2016; Molina-Alcaide et al. 2017). Coupled with a significant projected growth in the fisheries sector to meet a growing demand for protein (OECD/FAO 2015) and calls for the development of marine biomass within the blue growth initiative to support more sustainable bio-based economies (EU Commission 2012), the coming decades are likely to see significant increases in the development of off- and near-shore production systems, not just of seaweed, but also of fish, crustaceans and molluscs. Efforts are thus being directed to nurture a sustainable, low-impact and socially beneficial aquaculture industry (World Bank 2006; Gibbs 2009; Krause et al. 2015).

As detailed in Culver and Castle (2008) in numerous contributing case studies from Canada, coastal transformations such as the development of aquaculture in the wake of declining of fisheries can have significant implications for affected communities. Perceptions of aquaculture in Canada have been influenced by clashes with community values and further complicated by unpredictable aversion to innovation (Culver and Castle 2008). Given that studies have shown that perception of aquaculture seems to be linked to perceived environmental impacts (Katranidis et al. 2003; Whitmarsh and Wattage 2006), public perception of and potential opposition to aquaculture have been identified as an area of particular concern (Gibbs 2009; Schlag 2010; FAO 2015). However, on the whole, only a handful of studies have been conducted that look into perceptions of aquaculture among stakeholder groups, notably in New England (Robertson et al. 2002), Canada (Culver and Castle 2008; Barrington et al. 2010), Australia (Mazur and Curtis 2008), Spain (Bacher et al. 2014), Scotland (Whitmarsh and Palmieri 2009), Greece (Katranidis et al. 2003), a comparison between Germany and Israel (Freeman et al. 2012) and most recently two international (European) studies of stakeholder perceptions and acceptability of integrated multi-trophic aquaculture (Alexander et al. 2016a, b). Amongst these studies, a multitude of factors affecting perceptions are identified, ranging from awareness and knowledge levels, to credibility of information sources and environmental risks. Few of the studies, however, consider different types of aquaculture, and most assume the use of the generic term ‘aquaculture’ as pertaining exclusively to the culture of fish (with the exception of the last two mentioned above).

Significant differences in environmental performance between fed (e.g. finfish) and non-fed (e.g. seaweed and mollusc) aquacultures, resulting from different trophic positions of cultured species, have led to the assumption that there may be greater social acceptance of the latter, e.g. in Costa-Pierce (2010), though to the authors’ knowledge no studies have been conducted to validate this. There is also a lack of studies conducted on the perceptions of fed and non-fed aquacultures, and, most critically, on their perceived differences and associated concerns. The aim of this study is therefore to provide a baseline of current knowledge levels and awareness relating to aquaculture practices amongst residents of the Swedish West Coast, as a point of reference for future studies as aquaculture practices emerge and diversify on the West Coast. The study also aims to shed light on perceived differences between types of aquaculture likely to be developed in Sweden (fish, mollusc and seaweed) and their associated impacts, and to assess reactions to development scenarios of seaweed cultivation in view of identifying socio-oriented opportunities and risks.

## Materials and methods

**Fig. 1:**
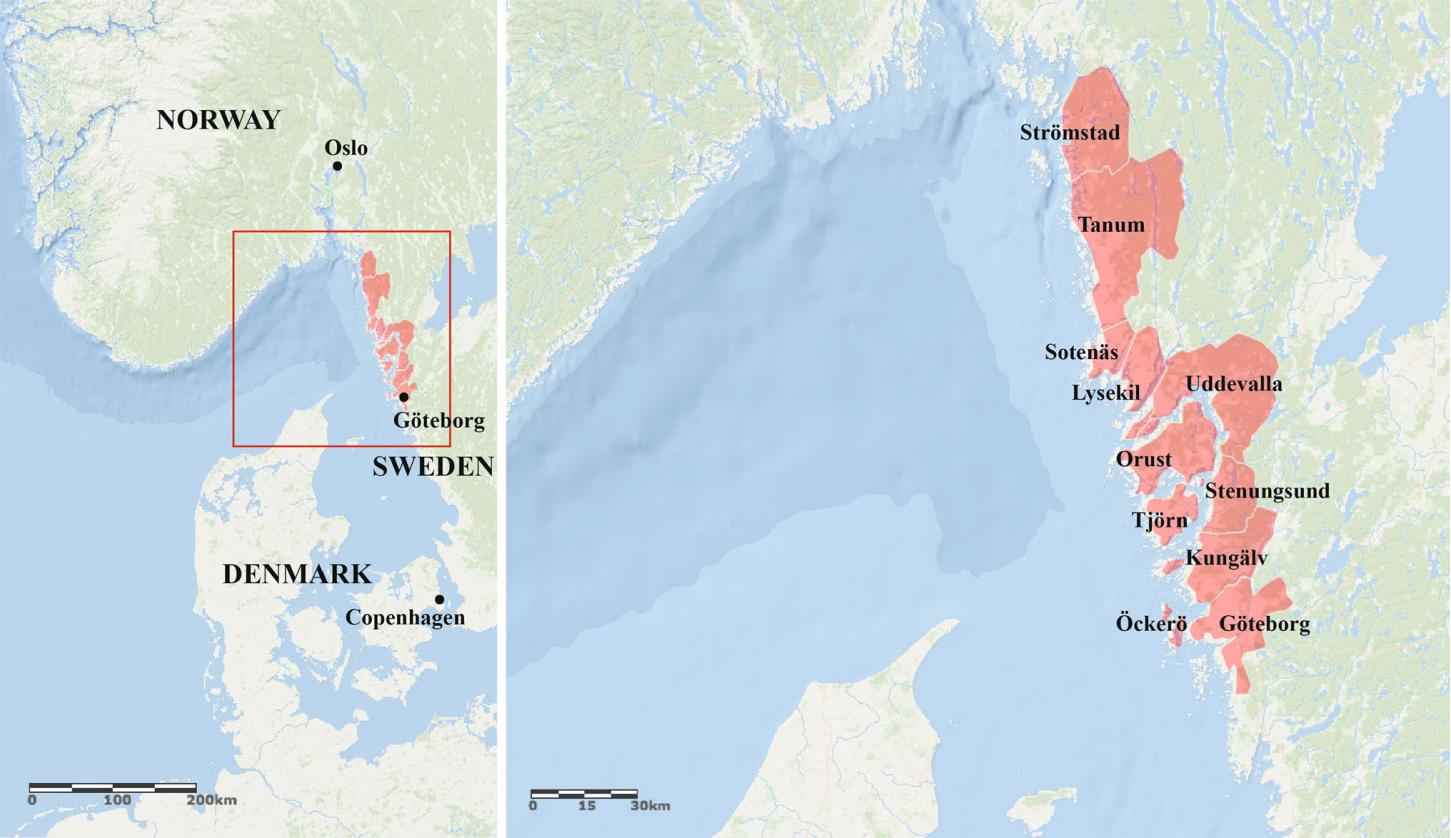
Map of study area highlighting the 11 municipalities targeted in the survey

A web-panel survey was conducted in 2015 with help of the fieldwork agency, Norstat. Members of the Norstat Panel with registered addresses in the study area (see Fig. 1) were randomly selected and offered financial compensation, SEK 40 (US $5), to respond to the online questionnaire. The survey was distributed in Swedish and translated to English for analysis. The responses from 695 respondents were included in the final analysis, from a total of 700 responses. To achieve a moderately representative sample from the residents of the West Coast, age and gender targets were set for each municipality to match the population of the study area using data from Statistics Sweden. Batches of invitations to participate in the survey were sent randomly to panel members over the months of July and August until the age and gender targets for each municipality were fulfilled. On the whole, the sample is considered to be representative of the population of the West Coast though respondents tended to show slightly lower than average incomes and marginally higher than average education qualifications.

The study area includes 11 municipalities (see Fig. 1) from the Västra Götaland region, selected for their tangency of the Skagerrak Sea, because of the presence of mussel aquaculture along this coastline, and also because of the likelihood that the area will see development of aquaculture in the coming decades, as these are among Sweden’s only territorial waters of non-brackish salinity. Furthermore, as a case study area for a perception survey, the West Coast is an ecotone of rich biodiversity and is considered nationally as an area of outstanding natural beauty, making it relevant and particularly sensitive to potential changes such as the development of blue growth initiatives, like seaweed aquaculture.

The questionnaire was designed in four parts, featuring questions requiring answers from a five point Likert scale including a middle/neutral option (e.g. very bad, bad, neutral, good, very good) or polar questions including a neutral option (e.g. yes, no, or don’t know). Some questions additionally offered discretionary comment sections. The first part of the survey aimed to provide ancillary information about respondents for subsequent use in statistical cross-referencing and analysis of patterns revealed by the main body of the survey. Their selection was based on authors’ knowledge of particularities of the region—location factors being considered important in studies of social acceptability (Freeman et al. 2012)—that may affect, or help to explain, specific attitudes toward aquaculture (e.g. the dichotomy between permanent residents and secondary holiday home owners, high levels of boat ownership, distance of property from the coast).

The second part of the questionnaire was the most extensive and sought to shed light on three key areas: (a) to assess aquaculture-related awareness levels and opinions toward aquaculture, including of different types of aquaculture and the differences between them; (b) to determine perceptions of five key aquaculture issues revolving around aesthetics and pollution; and (c) to gauge preliminary support for, or opposition to, the development of aquaculture on the West Coast.

The third part of the questionnaire presented some background information about the EU call for blue growth, coupled with a specific scenario for 2030 depicting the development of seaweed aquaculture on the West Coast and anticipated, associated changes, in an effort to determine reactions to this plausible future. A copy of the survey as seen by respondents is provided in the supplementary material 10.1007/s13280-017-0945-3. In light of the background information and the development scenario, respondents’ reactions were gauged and once again, they were asked about their support for or opposition to the development of aquaculture on the West Coast. The fourth and final part of the questionnaire covered basic information such as gender, age, education and income to the extent to which the sample could be considered representative of residents of the West Coast.

To explore the effect of knowledge levels and awareness on perceptions toward aquaculture, respondents were sorted into low, medium and high-awareness groups defined according to responses to a statement and a closed question (see Table 1). “No” responses for the statement placed respondents in the low-awareness group, “yes” responses to both questions placed respondents in the high-awareness group, and those who responded “yes” to the statement then “no” or “don’t know” to the question were placed in the medium-awareness group.

**Table 1:** Grouping of respondents by awareness levels according to answers to a question and a statement

Level of awareness	Low	Medium	High
Statement: “aquaculture may mean the cultivation of aquatic animals and/or plants. It depends”	“No”	“Yes”	“Yes”
Question: “are you aware of any differences in the farming of aquatic plants (seaweed), mollusks (mussels) and animals (fish), from an environmental point of view?”	–	“No”/“Don’t know”	“Yes”
Number of respondents	255	357	83
Percentage of sample	36.7	51.4	11.9

For statistical analysis of the results, an ordered probability model was used to test the relationship between perception (revealed via the Likert scale response variable) and a number of explanatory variables. The explanatory variables were selected to cover demographic and geographical variables, as suggested by Alexander et al. (2016b), as well as some additional factors the authors anticipated may have an effect based on their knowledge of the particularities of the region. These were as follows: distance between home address and coastline, visibility of the sea from respondents’ houses, the respondents’ aquaculture awareness, whether respondents go out to sea by boat, residence type (holiday house owner/permanent residence), awareness of a cultivation site near respondents’ homes, gender, education, age, income and the region that respondent lives in (or has a holiday house).

A similar statistical analysis has been undertaken by Alexander et al. (2016b) to analyse perception data of integrated multi-trophic aquaculture. The advantage of applying an ordered probability model, compared to the logit model in Alexander et al. (2016b) is that the former accounts for the natural order of the alternatives on the Likert scale in the estimation of the probabilities (see, e.g. Greene and Hensher 2010). The ordered probability model was built around the regression

1 $$ \gamma_{i}^{*} = \beta^{\prime} x_{i} + \varepsilon_{i} ,\quad i = 1, \ldots ,m, $$

where $\gamma_{i}^{*}$ is individual *i*’s stated option on the five point Likert scale (e.g. one of the alternatives very bad, bad, neutral, good, very good); the vector *x*_*i*_ is a set of explanatory variables; *β* is a vector of parameters to be estimated, and *ɛ*_*i*_ is the residual. For an overview of estimation and interpretation of ordered logit models, see, e.g. Greene and Hensher (2010), or Wooldridge (2010).

In the analysis, the 11 municipalities in Fig. 1 have been grouped into six different regions: (1) northern municipalities (Strömstad, Tanum, Sotenäs, Lysekil and Uddevalla), (2) islands (Orust, Tjörn and Öckerö), (3) middle municipalities (Stenungsund and Kungälv), (4) central Gothenburg, (5) areas north and south of central Gothenburg, (6) the most southern part of Gothenburg. Descriptive statistics for the explanatory variables are presented in Table 2.^1^

**Table 2:** Results from the ordered logit model: dependent variable general opinion toward aquaculture

Variables	Coefficients	Standard errors	*P*-values	Mean of the explanatory variable
Constant	5.16	0.55	0.00	
Distance home address and coastline	−0.04	0.07	0.48	2.82
Sea visible from home				
Yes	−0.06	0.22	0.80	0.24
No	0			0.76
Awareness				
High	0.59	0.26	0.02	0.12
Medium	0.13	0.16	0.43	0.51
Low	0			0.37
Go out to sea by boat				
Yes	0.47	0.19	0.01	0.23
No	0			0.77
Residence				
Holiday house owner	−0.74	0.34	0.03	0.06
Permanent resident	0			0.94
Cultivation sites near home				
Yes	0.52	0.19	0.01	0.22
No	0			0.88
Gender				
Female	−0.35	0.17	0.04	0.47
Male	0			0.53
Education				
Elementary school or high school <3 years	0			0.22
High school ≥3 years	−0.07	0.23	0.76	0.26
Higher education <3 years	0.50	0.22	0.03	0.24
Higher education ≥3 years	0.33	0.24	0.17	0.28
Region				
Islands (Orust, Tjörn and Öckerö)	−0.31	0.25	0.22	0.18
Areas north and south of central Gothenburg	−0.81	0.32	0.01	0.09
The most southern part of Gothenburg	−0.76	0.49	0.12	0.01
Central Gothenburg	−0.53	0.25	0.03	0.20
Northern municipalities	−0.57	0.22	0.01	0.33
Middle municipalities	0			0.19
Age^a^	0.17	0.05	0.00	5.19
Income	−0.07	0.06	0.23	2.79
Threshold parameter				
One	2.11	0.21	0.00	
Two	6.06	0.15	0.00	
Three	7.93	0.17	0.00	
Number of observations 695				

^a^Age is divided by 10. Northern municipalities (Strömstad, Tanum, Sotenäs, Lysekil and Uddevalla), middle municipalities (Stenungsund and Kungälv)

## Results

### Effects of awareness on perceptions of aquaculture

The results from the awareness sorting show that approximately a ninth of respondents qualified in the high-awareness group, half in the medium-awareness group and the remaining third in the low-awareness group.

**Fig. 2:**
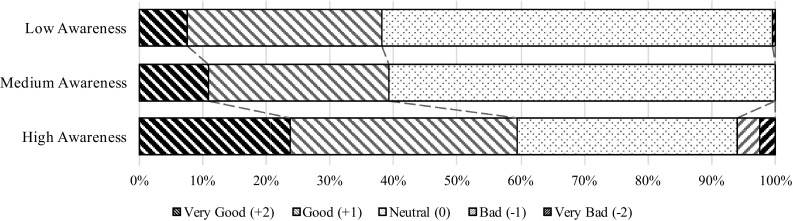
General opinions of aquaculture sorted by level of awareness

Overall analysis of results from all questions in the survey revealed some interesting awareness-related patterns that were consistently repeated throughout the survey (e.g. see Fig. 2). The low and medium-awareness groups showed similar responses, dominated by neutral responses on the five graded Likert scale, with neutral as the middle alternative. Higher proportions of neutral responses in the low- and medium-awareness groups confirm the notion that respondents in those groups were less informed on (or do not care about) aquaculture issues. The high-awareness group, while showing fewer neutral responses, tended to represent the same views as the low- and medium-awareness groups. General attitudes toward aquaculture were found not to significantly vary with awareness in this study; however, increased awareness did tend to lead to more pronounced opinions. A more thorough analysis of the respondents’ opinions is given in the next section.

### Perceptions of aquaculture

The focus of the survey was revealed to the respondents by the first question of part two, whereupon they were asked “how would you rate your general opinion toward aquaculture?” The results from this question are presented in Fig. 2 and sorted by awareness level. By selecting the neutral option, a majority of respondents demonstrated an initial tendency to be indifferent toward aquaculture and/or uninformed about aquaculture, but crucially, the rest of the respondents also tended to be favourable toward aquaculture rather than be opposed to it. In terms of awareness levels, the medium- and low-awareness groups showed almost identical results, with approximately 60% neutral/mid-scale responses and 40% rating their general opinions of aquaculture as either good or very good. This is in contrast to the opinions of respondents of the high-awareness group, a much smaller proportion of which selected neutral responses, and 25% and 35% of which selected ‘very good’ and ‘good’ ratings, respectfully. Also, a small number (less than 7%) of the high-awareness group selected the ‘bad’ and ‘very bad’ opinion responses.

The regression result for this question is presented in Table 2. In the ordered probit model, the dependent variable had the following distribution; very bad (*n* = 3), bad (*n* = 21), neutral (*n* = 391), good (*n* = 204), and very good (*n* = 76).

As seen from the table, most parameter estimates were statistically significant. The exceptions were as follows: distance between home address and coast line; whether the sea is visible from the respondents’ home (house/holiday house); and income.

According to the results in Table 2, individuals with high aquaculture awareness had a significantly more positive opinion toward aquaculture than individuals with a low level of awareness. The same result was found for individuals that had a cultivation site near their home, and individuals that go out to sea by boat. The sign of the point estimate must, however, be interpreted with caution, since it does not tell us how all cell probabilities (the probabilities that the individual’s state a specific alternative on the Likert scale) will be affected by a change in the explanatory variable. It is only for the first and last alternatives on the Likert scale (very bad and very good) that we can be sure about the sign of the change in the cell probability.

Table 3 reveals that the sign change in cell probabilities occurs between cells 2 and 3 (between neutral and good) for the explanatory variables in the model. Thus, a *positive* point estimate increases the probability of having a *good* or *very good* opinion toward aquaculture, whereas a *negative* point estimate increases the probability of having a *very bad*, *bad* or *neutral* opinion. However, as seen from Table 3, a negative point estimate mainly affects the probability of having a neutral opinion, whereas the marginal effect on the two lowest cells (very bad and bad) is much smaller.

**Table 3:** Marginal effects (in percentage units) on the probability that the respondent state a specific alternative on the Likert scale (very bad to very good), due to a change in the explanatory variable by one unit

Variables	Cells				
	0	1	2	3	4
	Very bad	Bad	Neutral	Good	Very good
Distance home address and coastline	0.02	0.11	0.97	−0.71	−0.38
Sea visible from home	0.02	0.14	1.18	−0.87	−0.46
High-awareness^a^	−0.17	−1.16	−13.22	8.59	5.97
Medium-awareness	−0.05	−0.31	−2.72	2.00	1.08
Go out to sea by boat^a^	−0.15	−1.01	−10.30	7.08	4.38
Holiday house owner^a^	0.37	2.46	13.27	−11.31	−4.79
Cultivation sites near home^a^	−0.16	−1.11	−11.52	7.82	4.96
Female^a^	0.12	0.84	7.30	−5.38	−2.89
High school ≥3 years	0.03	0.17	1.48	−1.09	−0.58
Higher education <3 years	−0.16	−1.08	−11.05	7.58	4.71
Higher education ≥3 years	−0.11	−0.75	−7.25	5.13	2.98
Islands (Orust, Tjörn and Öckerö)	0.12	0.82	6.30	−4.84	−2.40
Areas north and south of central Gothenburg^a^	0.40	2.66	14.30	−12.17	−5.19
The most southern part of Gothenburg	0.40	2.62	13.25	−11.52	−4.75
Central Gothenburg^a^	0.22	1.48	10.39	−8.19	−3.90
Northern municipalities^a^	0.22	1.51	11.55	−8.84	−4.45
Age^a,b^	−0.06	−0.42	−3.67	2.70	1.45
Income	0.02	0.17	1.48	−1.08	−0.58

1.0 Denotes a change in the probability of one percentage point

^a^Denotes that the estimated coefficient in the ordered probit model was significant at a 5% significance level

^b^The marginal effect represents a change in age with 10 years

The largest marginal effects were found for groups of individuals with a high aquaculture awareness and for holiday house owners. Compared to permanent residents, holiday house owners have 11 percentage units lower probability for having positive opinions, and 13 percentage units higher probability for having a neutral opinion toward aquaculture.

Concerning the regional variable, individuals living in the reference region (the middle municipalities: Stenungsund and Kungälv) have the most positive opinion toward aquaculture. People living in the northern municipalities, central Gothenburg and in areas north and south of central Gothenburg have a significantly lower probability of stating a *good* or *very good* opinion towards aquaculture, compared to groups of individuals living in the reference region. The probability for stating a good opinion is about 9 percentage units lower. Individuals living in the northern municipalities, central Gothenburg and in areas north and south of central Gothenburg, have instead a more neutral opinion towards aquaculture. These findings may be another example of the importance of location, specifically rural and urban locations, in the variability of perceptions toward aquaculture as identified by Katranidis et al. (2003).

There is no significant difference in the opinions toward aquaculture for groups of individuals living on the islands (Orust, Tjörn and Öckerö) and groups of individuals living in the reference region (Stenungsund and Kungälv). These islands are also located close to the reference region.

The results also suggested that there is a significant difference between women and men in their general opinion toward aquaculture, where men are more positive than women. Older people also had a more positive opinion toward aquaculture compared to younger people. The marginal effects for the gender and age variables are smaller than for other statistically significant variables.

### Perceptions of different types of aquaculture

**Fig. 3:**
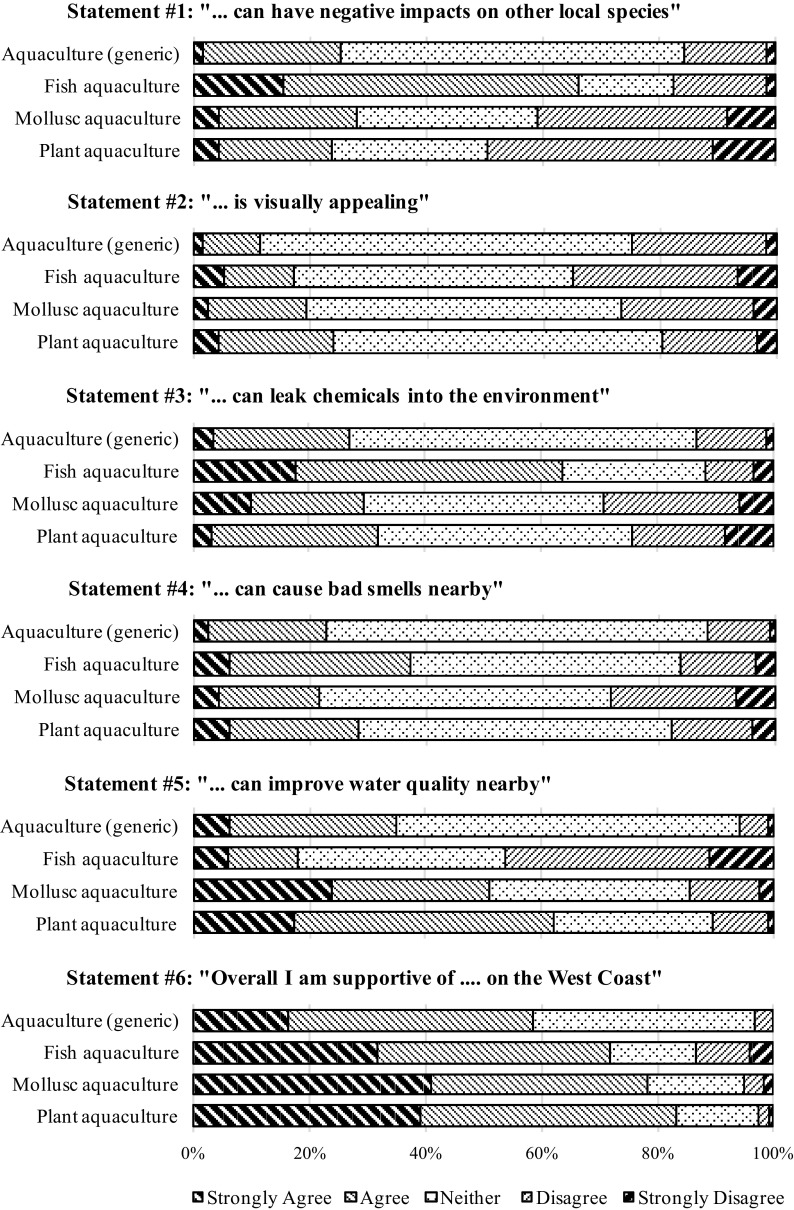
Reactions to six statements regarding fish, mollusc, plant and generic aquaculture

Following this initial exposure to aquaculture, respondents were asked “Are you aware of any differences in the farming of aquatic plants (seaweed), molluscs (mussels) and animals (fish), from an environmental point of view?”. 17% of respondents answered that they were aware of differences between different types of aquaculture, while 83% were not aware of any differences. Those unaware of differences were provided with six statements about generic aquaculture only, whereas those aware of differences were provided with the same six statements but separately for each seaweed, mollusc and fish aquaculture. The responses to these six statements—for each generic aquaculture, fish aquaculture, seaweed aquaculture and mollusc aquaculture—are presented in Fig. 3.

A series of key results should be highlighted from Fig. 3. First, the “neither” agree nor disagree option is on average the most prevalent across all statements. Notably, it is systematically larger in the responses for generic aquaculture (always above 59% of respondents, excepting Statement 6), compared to those for fish, mollusc and plant aquaculture. This could be a sign that, as a whole, respondents are not sufficiently acquainted with aquaculture issues to have well-formed opinions. Second, when comparing aquaculture types, responses reflected that mollusc and plant aquaculture are perceived as being quite similar to one another, but quite different from fish aquaculture. This is with the exception of Statements 2 and 4, regarding the visual aesthetics and potential for bad smells, respectively, for which all aquaculture types performed similarly with large neutral fractions and balanced opinions across the sample. Fish aquaculture was perceived as having much more potential to have negative impacts on other local species and to leak chemicals into the environment (e.g. feed), when compared to mollusc, plant and generic aquaculture. For Statement 5, 46% of respondents disagreed with the statement that fish aquaculture could improve water quality, however 51 and 62% of respondents agreed that mollusc and plant aquaculture (respectively) could improve water quality.

In spite of the various concerns emphasised by responses to the previous statements, Statement 6 revealed a significant inclination for respondents to be supportive of all of the aquaculture types on the West Coast. A slight preference for mollusc and seaweed was also clear, while fish aquaculture showed the most opposition of the four options, and generic aquaculture saw more neutral responses than the other types. Finally, it should be noted that the responses regarding generic aquaculture were quite similar to those for mollusc and plant aquaculture on the whole.

### Aquaculture development scenarios on the West Coast

The third part of the questionnaire began by presenting some background information, introducing respondents to the EU bioeconomy strategy and the need for renewable biological resources, notably marine ones, to secure sustainable economic growth. Thereafter, a scenario was presented depicting a future for the Swedish West Coast, whereby in 2030 there would be seaweed aquaculture sites spread along the coast, covering a total area of approximately 10 km^2^, both providing some ecosystem services and biomass for biorefineries and thus employment opportunities and incomes for the region, but also having some unknown environmental impacts on the sea bed. See supplementary information 10.1007/s13280-017-0945-3 for a copy of the survey as seen by respondents.

A large majority of respondents were favourable toward the depicted scenario: 14 and 48% of respondents were very positive and positive, respectively, while 6% selected the negative option and only one respondent (out of 695) chose the very negative option. Respondents were, however, of mixed opinions when asked about their scepticism of the economic and environmental claims portrayed in the scenario, with notable variation across the awareness groups. Approximately 30% of each awareness group confirmed they were sceptical about the claims. However, there is a shift from mostly neutral responses in the low and medium-awareness groups to a tendency for the high-awareness group to trust the scenario claims: while the low and medium-awareness groups had between 40 and 50% selecting the neutral responses, almost 50% of the high-awareness group disagreed or strongly disagreed with the statement that they were sceptical of the portrayed claims.

**Fig. 4:**
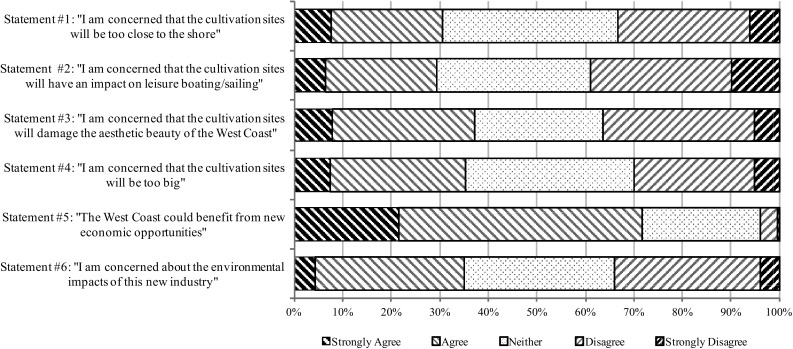
Key concern statements about the described scenario

To further explore reactions to the scenario, respondents were asked whether they agreed or disagreed (also on a five point Likert scale, with a neutral option) to six statements representative of key areas of concern. The results are presented in Fig. 4. Overall responses were more or less evenly distributed for each statement, with approximately equal numbers agreeing and disagreeing to each statement and with large portions selecting the neutral options. Once again this may be a sign that residents of the West Coast are not sufficiently informed about aquaculture issues to have well-formed and consistent opinions. However, the fifth statement was found to be the exception: 22% of respondents strongly agree and 50% agree with the statement that “the West Coast could benefit from new economic opportunities”.^2^

Ordered logit models with the same set of explanatory variables as in Eq. (1) has also been estimated for the six statements in Fig. 4. Most point estimates in these regressions where insignificant, with the exception of the gender and age variables that turned out to be statistical significant at a 5% significance level (*P* value <0.05). The point estimate for the gender variable was negative, which suggests that female respondents were more concerned than males across the six concern statements of Fig. 4. The point estimate for the age variable was positive, which indicates that older individuals were less concerned than younger individuals across the six statements.

The final question of part three of the survey, relating to the scenario description, asked respondents: “Would you say that you would be supportive of such blue-growth developments?” with only yes and no as answer options. On average, four out of five respondents (78%) expressed that yes, they were supportive of such blue growth initiatives, with the high-awareness group showing an even stronger majority (89%). These results suggest that West Coast residents, on the whole, may have some scepticism toward the benefit claims and lingering concerns regarding the potential impacts of seaweed aquaculture, but nevertheless, a consistent majority are supportive of its development.

## Discussion and conclusion

### Awareness

Throughout the survey, opinions of the high-awareness group were found to be marginally stronger due to that group being less prone to select neutral responses. This seems an indication that opinions of these respondents are more developed than those of the lower awareness groups, which also acts as a validation of the efficacy of awareness categorisation applied in this study. Furthermore, given the relatively more favourable perceptions toward aquaculture expressed by the high-awareness group, it may also indicate that increased education and regular communication with stakeholders of aquaculture (defined in the broadest of terms) could improve the acceptability of aquaculture. This resonates within literature where similar studies have supported that effective communication and increasing education about aquaculture can improve its social acceptability (Kaiser and Stead 2002; Robertson et al. 2002; Barrington et al. 2010).

The large fraction of consistently neutral responses that represent individuals who may be uninformed and/or indifferent toward aquaculture, particularly in the low and medium-awareness groups, may be regarded a potential threat to social acceptability in the future (Robertson et al. 2002). That a majority of respondents may be uninformed and/or indifferent toward aquaculture is also consistent with other aquaculture perception studies, such as the pan-European perceptions study by Alexander et al. (2016b) and that conducted by Barrington et al. (2010) in Canada. Social aversion to innovation is notoriously unpredictable, though as raised by Culver and Castle (2008), it is thought that it can be particularly strong when the beneficiaries of this innovation are not aware of, or do not need, said benefits. In the case of this study however, it would seem that the benefits, particularly the regeneration of the West Coast through economic opportunities and environmental improvements, are desirable for now and thus may be generating part of the support evident in the results in spite of the large neutral fraction. Increasing and maintaining awareness on the benefits of sustainable aquaculture practices—coupled with vigilant monitoring of aquaculture’s social impacts and its perceived value—will be essential for a healthy relationship between aquaculture on the West Coast and the people who live there.

### Types of aquaculture and impacts

The perceived differences between fish, plant and mollusc aquaculture by the high-awareness group, with the added comparison to perceptions of generic aquaculture of the medium and low-awareness groups, are some of the key highlights revealed in this study. In ecological terms, plants, molluscs and fish belong to different levels of the classic trophic pyramid, each characterised by different relationships with their shared ecosystem, notably in terms of the flows of energy and nutrients through the food chain. Increasing the population of a species from one trophic level, for instance by conducting finfish aquaculture, can change a local ecosystem. This study identified that respondents who were aware of different types of aquaculture also showed a tendency to be aware of associated impacts. The perceptions of fish aquaculture are clearly contrasting to those of plant and mollusc aquaculture, as seen in Statements 1 and 5 from Fig. 3, respectively concerned with impacts on other local species and the improvement of water quality (i.e. classic environmental impact and ecosystem service). Whereas the trend for seaweed and mollusc aquaculture was for respondents to disagree that they have impacts on other local species and to agree that they could improve water quality, the exact opposite was true for fish aquaculture. This may both be a reflection that many of these respondents are aware of these different trophic roles, but also of the relatively high impacts of the fish aquaculture industry. This latter aspect, the perceived high impacts of fish aquaculture, is echoed in the results of Statement 3 wherein fish aquaculture was thought of as having a high potential to leak chemicals into the environment (e.g. feed), whereas respondents were more balanced and/or indecisive regarding the potential for chemical leakage in mollusc and plant aquaculture. These results are in line with similar findings in literature, for instance in Alexander et al. (2016b).

Finally, the responses to Statement 6 carry particular significance. Though not an example of the value-action gap per se, this is similar and could be said to exemplify a perception-support gap: in spite of a clearly negative perception of one option, all options are given similar support. While fish aquaculture received slightly less support than mollusc and plant aquaculture, given the high perceived environmental risks associated to it, one might have expected more opposition. In the next section, a key potential reason for this support is identified.

### Looking forward

As a whole, it would seem that the perceived environmental aspects of different aquaculture types, though clearly important factors affecting support for or aversion to aquaculture, represent only relatively minor influences. The much greater factor at play here, as seen in Fig. 4, is the potential for economic betterment of the West Coast by developing aquaculture. This is a significant finding, revealing a key popular pressure—the popular desire for more economic opportunities—in the drive to develop aquaculture on the Swedish West Coast. These views are further reinforced by the support expressed by respondents for the scenario portrayed in the survey, which depicts further development of seaweed aquaculture on the West Coast in the coming years.

It is also clear from Fig. 4 that respondents were of mixed opinions regarding some key concerns such as the aesthetic and environmental impacts of the cultivations described in the scenario, contrary to what the authors had anticipated. For instance, it had been expected that there would be significant opposition from respondents who go to sea regularly due to the farms occupying valued sea space, yet those respondents were statistically less likely to be opposed or neutral and more likely to be supportive of aquaculture (see Table 3). On the whole, there was a lack of specific opposition about impacts on leisure boating (see Statement 2 of Fig. 4). On the other hand, both age and gender variables were found to be statistically significant in their effect on responses to the areas of concern presented in Fig. 4, though seemingly in contradiction to other studies (Fernandez-Polanco et al. 2008): older respondents showed less concern across the six statements than younger respondents, while gender was found to show no effect in previous studies. Possible reasons for these differences are unclear; however, it should be noted that though both of these studies pertain to perceptions of aquaculture, each focuses on different types of aquaculture. Furthermore, opinions and perceptions of aquaculture will change over time and should be re-evaluated in the future, particularly as aquaculture infrastructure becomes more common and obstructs larger spaces of the West Coast.

In addition, a large number of respondents were sceptical towards some of the other claims made in the scenarios. This again exemplifies the aforementioned perception-support gap, possibly resulting from a desire for more economic opportunities, whereby a majority of respondents remained favourable to the notion of more aquaculture on the West Coast in spite of being divided on a range of issues and while being sceptical of the scenario. This scepticism and division of opinion, but especially the minority of respondents who were opposed to aquaculture developments on the West Coast, represent important potential risks to a stable development of aquaculture on the West Coast. They highlight the need to raise awareness, particularly about impacts, how aquaculture developments will affect individuals, the potential for generating work in the region and on the ecosystem services of sustainable aquaculture practices.

As seen with the controversy surrounding the carrageenan industry (Bixler 2017), an important portion of the global seaweed industry, hostility to the seaweed industry has been—and can be—rapidly mobilised on a global scale by a minority of opposed individuals, in spite of scientific evidence refuting the hostile claims (McKim 2014; Weiner 2014). Further research should be undertaken to ascertain reasons for opposition to aquaculture on the West Coast and to pre-emptively identify solutions.

The complexity of aquaculture practices and the unintended consequences of their development are known to contribute to social aversion to aquaculture, as documented in extensive contributions in Culver and Castle (2008) relating to a range of issues such as the social transformations experienced by coastal communities in Canada. There are lessons to be learnt from such cases. By providing a benchmark of current perceptions toward aquaculture on the Swedish West Coast, it is hoped that this study may provide valuable information to policy makers and industry to avoid mistakes made elsewhere (like in Canada), but also as a point of reference for future studies of social aversion toward aquaculture. It should not be assumed, however, that the support for seaweed aquaculture development scenarios revealed by this study will be maintained. Location factors are considered important in surveys of social acceptability (Freeman et al. 2012). The results of this survey are a unique snapshot of attitudes toward aquaculture on the Swedish West Coast in 2015 and attitudes may not be the same in 10 years. As such, the authors assert that there is a genuine need for systematic monitoring of potential drivers and barriers, as proposed by Krause et al. (2015), for a more transparent, socially, environmentally and economically sustainable development of seaweed aquaculture on the West Coast.

## Electronic supplementary material

Below is the link to the electronic supplementary material.

Supplementary material 1 (PDF 604 kb)

## Footnotes

1 The region where the respondents have the most positive attitudes for aquaculture will be treated as the reference region in the ordered logit model, to facilitate the interpretation of the results.

2 Whereas the five other statements covering key areas of concern all specifically refer to aquaculture, it should be noted that this statement does not. However, given that the other statements are in reference to aquaculture, it is assumed that respondents frame the context of this statement accordingly.
