# Supplementary material for: The perception of aquaculture on the Swedish West Coast
Source: Ambio. 2017 Sep 22;47(4):398–409. doi: 10.1007/s13280-017-0945-3 (PMC5884760; doi:10.1007/s13280-017-0945-3)
Supplement: Supplementary file 1 — Supplementary material 1 (PDF 604 kb) [file 13280_2017_945_MOESM1_ESM.pdf]

Ambio

Electronic Supplementary Material
This supplementary material has not been peer reviewed.

Title: The perception of aquaculture on the Swedish West Coast

Authors: Jean-Baptiste E. Thomas, Jonas Nordström, Emma Risén, Maria E. Malmström, Fredrik Gröndahl

Välkommen och tack för att du tar dig tid till att svara på den här undersökningen.

Du har valts ut till att vara en av 700 deltagare i den här undersökningen angående uppfattningar om vattenbruk i Västra Götalandsregionen. Dina åsikter och uppfattningar är viktiga för oss även om du inte vistas regelbundet i detta område eller har någon särskild kunskap om vattenbruk.

Undersökningen genomförs av KTH i Stockholm samt Lunds Universitet i samarbete med Chalmers, Göteborgs Universitet och Linnéuniversitetet.

Alla dina svar kommer att behandlas anonymt och kommer inte att spåras tillbaka till dig.

Undersökningen bör ta cirka 10 till 15 minuter att genomföra.

- -

1. Observera att du inte kan gå tillbaka och ändra ett svar efter det att du har gått vidare till nästa fråga.

2. Vilket eller vilka påstående(n) beskriver dig bäst?

- ☐ Jag bor i en kommun vid västkusten permanent
- ☐ Jag har en semesterbostad i en kommun vid västkusten
- ☐ Om annat, var god att specificera

3. Skriv din postkod i utrymmet nedan

4. Hur långt från kusten är din bostad belägen?

5. Är havet synligt från din bostad?

- Ja
- Nej

6. Har du direkt tillgång till en båt på västkusten? (egen, delad eller familjebåt)

- Ja
- Nej

7. För varje årstid ange ungefärligt hur ofta du tar båten ut till havs.

	Oftare än en gång i veckan (mycket regelbundet)	En gång i veckan (regelbundet)	En gång i månaden (emellanåt)	Inte alls
Vår (mars, april, maj)				
Sommar (juni, juli, augusti)				
Höst (september, oktober, november)				
Vinter (december, januari, februari)				

8. Ägnar du dig åt någon speciell aktivitet när du åker ut med din båt? (t.ex. fiske, dykning, vindsurfing och/eller annan vattensport eller vattenbaserad aktivitet)

- Ja
- Nej

Om ja, var god ange vilken aktivitet.

9. VATTENBRUK: övergripande uppfattningar

Du kommer inom kort att få en presentation av en av de allmänt accepterade definitionerna av vattenbruk.
Innan dess vill vi dock utforska din nuvarande förståelse av ordet, baserat på dina befintliga kunskaper.

10. Vad tycker du om vattenbruk i allmänhet?

- Mycket dåligt
- Dåligt
- Neutralt inställd
- Bra
- Mycket bra

Det är mycket intressant för oss att ta reda på hur du tänker kring vattenbruk, även om du egentligen inte har en stark åsikt i frågan. Vänligen ta en stund till att förklara ditt svar på föregående fråga och dela med dig av dina tankar och kommentarer nedan.

11. Ange i vilken utsträckning du instämmer eller inte instämmer med vart och ett av följande påståenden:

	Instämmer helt	Instämmer till viss del	Har ingen åsikt	Instämmer inte	Instämmer inte alls
Vattenbruk är dåligt för miljön	○	○	○	○	○
Vattenbruksanläggningar är estetiskt oattraktiva	○	○	○	○	○
Det bör finnas mer vattenbruk på västkusten	○	○	○	○	○

12. Känner du till några odlingsplatser för vattenbruk nära ditt hem?

○ Ja
○ Nej

13. Kryssa i rutan eller rutorna för det eller de påståenden som du tror är korrekta.

☐ Vattenbruk är som jordbruk, men i vattnet.
☐ Vattenbruk är endast odling av vattenlevande djur, såsom fisk, musslor eller krabbor.
☐ Vattenbruk är endast odling av vattenlevande växter, såsom alger.
☐ Vattenbruk kan bestå av odling av vattenlevande djur och växter. Det beror på.

14. En av de allmänt accepterade definitionerna av vattenbruket är:

Uppfödning av vattenlevande djur eller odling av vattenlevande växter för livsmedel.

15. Är du medveten om eventuella skillnader mellan odling av vattenväxter (alger), blötdjur (musslor) och djur (fisk), ur miljösynpunkt?

○ Ja
○ Nej
○ Jag vet inte

16. Följande uttalanden avser miljöeffekterna av vattenbruk och syftar till att belysa hur du tror att VÄXT-, FISK- för BLÖTDJURvattenbruk skiljer sig åt i fråga om miljöpåverkan.

	Instämmer helt	Instämmer till viss del	Har ingen åsikt	Instämmer inte	Instämmer inte alls
**Växtvattenbruk** har negativa effekter på andra arter i sin **närmiljö**	○	○	○	○	○
**Fiskvattenbruk** har negativa effekter på andra arter i sin **närmiljö**	○	○	○	○	○
**Blötdjurvattenbruk** har negativa effekter på andra arter i sin **närmiljö**	○	○	○	○	○
**Växtvattenbruk** är **visuellt tilltalande**	○	○	○	○	○
**Fiskvattenbruk** är **visuellt tilltalande**	○	○	○	○	○
**Blötdjurvattenbruk** är **visuellt tilltalande**	○	○	○	○	○
**Växtvattenbruk** kan läcka **kemikalier** i miljön	○	○	○	○	○
**Fiskvattenbruk** kan läcka **kemikalier** i miljön	○	○	○	○	○
**Blötdjurvattenbruk** kan läcka **kemikalier** i miljön	○	○	○	○	○
**Växtvattenbruk** kan orsaka **dålig lukt** i sin närhet	○	○	○	○	○
**Fiskvattenbruk** kan orsaka **dålig lukt** i sin närhet	○	○	○	○	○
**Blötdjurvattenbruk** kan orsaka **dålig lukt** i sin närhet	○	○	○	○	○
**Växtvattenbruk** kan förbättra **vattenkvaliteten** i sin närhet	○	○	○	○	○
**Fiskvattenbruk** kan förbättra **vattenkvaliteten** i sin närhet	○	○	○	○	○
**Blötdjurvattenbruk** kan förbättra **vattenkvaliteten** i sin närhet	○	○	○	○	○

17. Följande uttalanden avser miljökonsekvenserna av vattenbruk.

	Instämmer helt	Instämmer till viss del	Har ingen åsikt	Instämmer inte	Instämmer inte alls
Vattenbruk har negativa effekter på andra arter i sin **närmiljö**	◯	◯	◯	◯	◯
Vattenbruk är **visuellt tilltalande**	◯	◯	◯	◯	◯
Vattenbruk kan läcka **kemikalier** i miljön	◯	◯	◯	◯	◯
Vattenbruk kan orsaka **dålig lukt** i sin närhet	◯	◯	◯	◯	◯
Vattenbruk kan förbättra **vattenkvaliteten** i sin närhet	◯	◯	◯	◯	◯

18. Följande uttalanden relaterar till din allmänna uppfattning om växt-, fisk- och blötdjurvattenbruk på västkusten.

	Instämmer helt	Instämmer till viss del	Har ingen åsikt	Instämmer inte	Instämmer inte alls
På det stora hela stödjer jag växtvattenbruk på västkusten.	◯	◯	◯	◯	◯
På det stora hela stödjer jag fiskvattenbruk på västkusten.	◯	◯	◯	◯	◯
På det stora hela stödjer jag blötdjurvattenbruk på västkusten.	◯	◯	◯	◯	◯

19. Följande uttalande avser din allmänna uppfattning om vattenbruk på västkusten.

	Instämmer helt	Instämmer till viss del	Har ingen åsikt	Instämmer inte	Instämmer inte alls
På det stora hela stödjer jag vattenbruk på västkusten.	◯	◯	◯	◯	◯

20. Finns det några andra typer av miljökonsekvenser som du känner till och vill nämna?

◯ Ja
◯ Nej

Om ja, var god och ange dessa.

Det är troligt att en algindustri kommer att utvecklas på västkusten under nästa decennium eller så. I nästkommande del av undersökningen kommer algindustrin beskrivas och kortfattat förklaras i form av ett framtidsscenario. Baserat på detta framtidsscenario kommer du att bli tillfrågad om dina åsikter och synpunkter.

Var god ta din tid till att läsa följande information och begrunda bilden samt kartan.

BAKGRUND:

I februari 2012 antog Europeiska kommissionen en strategi för att leda den europeiska ekonomin mot en mer storskalig och hållbar användning av förnybara resurser. Programmet benämns "**Innovating for Sustainable Growth: a Bioeconomy for Europe**". Världens befolkning närmar sig 9 miljarder år 2050 och många av våra naturresurser är ändliga. Europa är därför i behov av förnyelsebara biologiska resurser för att säkerställa tillgång på matvaror, foder, material, energi och andra produkter.

Som ett led i denna strävan att utveckla ny teknik för förnybara biologiska resurser, har en del innovatörer vänt sina blickar mot haven och kusterna och utforskat dem som nya potentiella plattformar för hållbar ekonomisk tillväxt. Ett samlingsnamn för detta är **Blue Growth**. Odling av vattenlevande växter och djur liksom alger, musslor, sjögurkor och många andra arter tros kunna gynna sin närmiljö. Denna typ av odling i havsmiljö granskas för närvarande intensivt för att förstå de potentiella effekter storskaliga odlingar kan få, för att identifiera potentiella produkter man kan utvinna och undersöka marknaden för dessa samt för att få en uppfattning om hur detta kan bidra till den europeiska bioekonomin.

SCENARIO:

Vi befinner oss nu under år 2030 och en del större förändringar har skett på västkusten sedan 2015.

Ett antal odlingsplatser längs Skagerraks och Kattegats kuster producerar alger och andra producerar marin biomassa till en nu väletablerad havsbaserad industri. Ett exempel på en sådan odlingsplats kan ses i Figur 1. Odlingsplatserna representeras av röda rektanglar i figur 2, och upptar lite mindre än 1% av det totala kustområdet, vilket totalt motsvarar 1000 hektar (10km2) havsyta.

Odlingsplatserna tros ha en positiv inverkan på miljön, genom att absorbera en del av det överskott av näringsämnen som forslas från land ut i havet. Vattenkvaliteten har synbart förbättrats, liksom mångfalden av marint liv i området. Havsbottnen har dock förändrats som ett resultat av skuggning från odlingen. Mer forskning behöver göras för att förstå dessa förändringar.

Produkterna från odlingarna bearbetas till hållbart producerade varor i stora bioraffinaderier längs kusten, vilket ger arbete åt flera hundra personer och genererar inkomster till regionen.

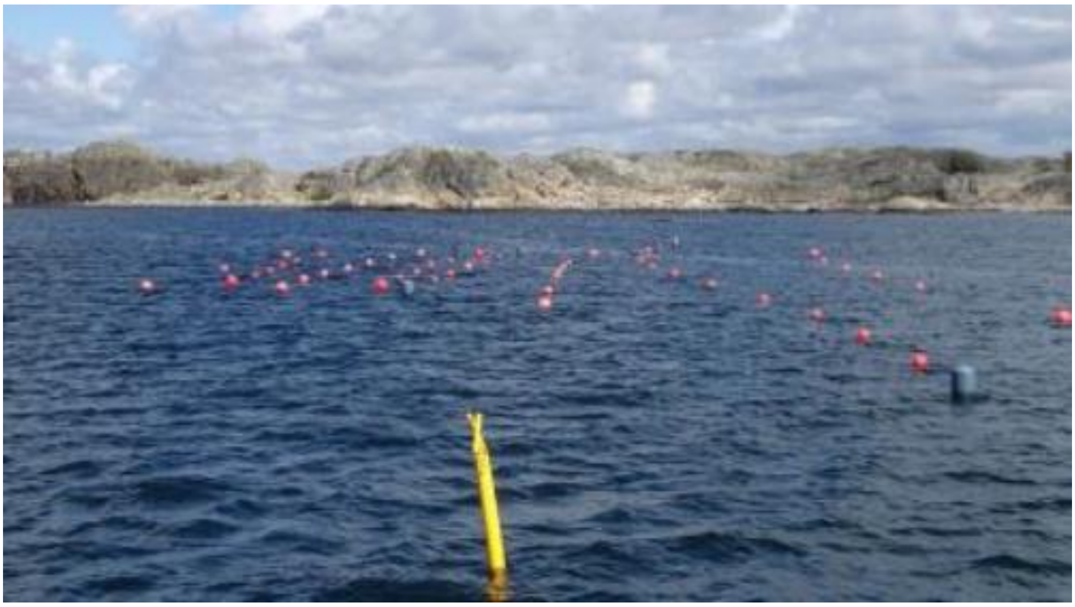

Figur 1: En liten algodling i Koster skärgård (maj 2015). Gårdarna i scenariot kommer att vara mycket större.

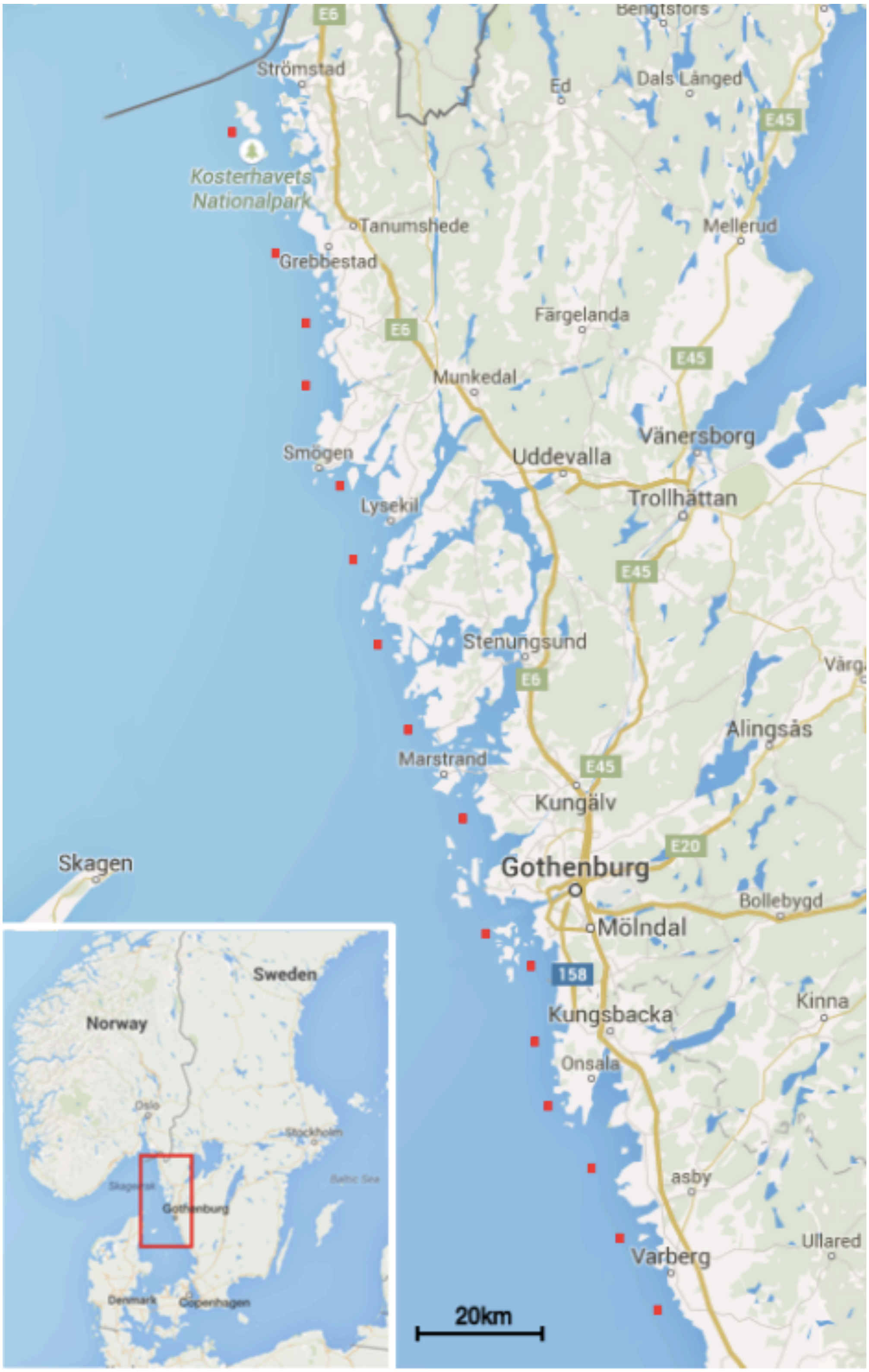

Figur 2: Jämnt fördelade odlingsplatser (i rött) i ett scenario för västkusten år 2030. De röda kvadraterna är ej skalenliga men representerar lokalisationen av föreslagna odlingsplatser.

21. Vad är din första reaktion på detta scenario?

- Mycket positiv
- Positiv
- Neural
- Negativ
- Mycket negativ

22. Ange i vilken utsträckning du instämmer med eller tar avstånd från följande påståenden:

	Instämmer helt	Instämmer till viss del	Har ingen åsikt	Instämmer inte	Instämmer inte alls
Jag känner mig skeptisk till påståendena kring miljöpåverkan i detta scenario.	○	○	○	○	○
Jag känner mig skeptisk till påståendena kring de ekonomiska effekterna i detta scenario	○	○	○	○	○

23. Ange i vilken utsträckning du instämmer med eller tar avstånd från följande påståenden:

	Instämmer helt	Instämmer till viss del	Har ingen åsikt	Instämmer inte	Instämmer inte alls
Jag är bekymrad över miljökonsekvenserna kopplade till denna nya bransch	○	○	○	○	○
Västkusten skulle kunna dra nytta av nya ekonomiska möjligheter	○	○	○	○	○
Jag är bekymrad över att odlingsplatserna kommer att vara för stora	○	○	○	○	○
Jag är bekymrad över att odlingsplatserna kommer att skada västkustens (visuella/estetiska) skönhet.	○	○	○	○	○
Jag är bekymrad över att odlingsplatserna kommer att påverka användningen av fritids- och segelbåtar	○	○	○	○	○
Jag är bekymrad över att odlingsplatserna kommer att vara belägna alltför nära land	○	○	○	○	○

24. Stödjer du en sådan utveckling av marin tillväxt?

○ Ja
○ Nej

Var god lista dina främsta skäl.

25. Sex alternativa användningsområden för havet finns listade nedan. Föreställ dig att ett havsområde nära kusten tilldelas ett av dessa alternativ. Bedöm nu dessa användningsområden genom att ge dem en siffra från 1 till 5 (där 5 är att föredra och 1 är det minst föredragna alternativet)

	1 (Minst föredragna)	2	3	4	5 (Att föredra)
Inget nyttjande av havet	○	○	○	○	○
Vattenbruk med odling av alger	○	○	○	○	○
Vindkraftpark	○	○	○	○	○
Fiskeområde	○	○	○	○	○
Kombinerad vattenbruk (alger, fisk och musslor, till exempel)	○	○	○	○	○
Vågenergigård	○	○	○	○	○

Innan du avslutar undersökningen, skulle vi vilja ställa några ytterligare korta frågor som följer:

26. Jag är

27. Födelseår (åååå)

28. Högsta utbildningsnivå

29. Månadsinkomst i SEK (efter skatt)

30. Har du hemmaboende barn (under 18)?

- ○ Ja
- ○ Nej

31. Hur många personer finns i hushållet (med dig inkluderad)?

Var god skicka in dina svar.
